# PIF4 Promotes Expression of *LNG1* and *LNG2* to Induce Thermomorphogenic Growth in *Arabidopsis*

**DOI:** 10.3389/fpls.2017.01320

**Published:** 2017-07-25

**Authors:** Geonhee Hwang, Jia-Ying Zhu, Young K. Lee, Sara Kim, Thom T. Nguyen, Jungmook Kim, Eunkyoo Oh

**Affiliations:** ^1^Department of Bioenergy Science and Technology, Chonnam National University Gwangju, South Korea; ^2^Department of Plant Biology, Carnegie Institution for Science, Stanford CA, United States; ^3^Cold Spring Harbor Laboratory, Cold Spring Harbor NY, United States; ^4^Division of Biological Sciences and Institute for Basic Science, Wonkwang University Iksan, South Korea

**Keywords:** Arabidopsis, high temperature stress, thermomorphogenesis, PIF4, LNG, gene expression, ChIP

## Abstract

*Arabidopsis* plants adapt to high ambient temperature by a suite of morphological changes including elongation of hypocotyls and petioles and leaf hyponastic growth. These morphological changes are collectively called thermomorphogenesis and are believed to increase leaf cooling capacity by enhancing transpiration efficiency, thereby increasing tolerance to heat stress. The bHLH transcription factor PHYTOCHROME INTERACTING FACTOR4 (PIF4) has been identified as a major regulator of thermomorphogenic growth. Here, we show that PIF4 promotes the expression of two homologous genes *LONGIFOLIA1* (*LNG1*) and *LONGIFOLIA2* (*LNG2*) that have been reported to regulate leaf morphology. ChIP-Seq analyses and ChIP assays showed that PIF4 directly binds to the promoters of both *LNG1* and *LNG2*. The expression of *LNG1* and *LNG2* is induced by high temperature in wild type plants. However, the high temperature activation of *LNG1* and *LNG2* is compromised in the *pif4* mutant, indicating that PIF4 directly regulates *LNG1* and *LNG2* expression in response to high ambient temperatures. We further show that the activities of LNGs support thermomorphogenic growth. The expression of auxin biosynthetic and responsive genes is decreased in the *lng* quadruple mutant, implying that LNGs promote thermomorphogenic growth by activating the auxin pathway. Together, our results demonstrate that *LNG1* and *LNG2* are directly regulated by PIF4 and are new components for the regulation of thermomorphogenesis.

## Introduction

Increases in ambient temperature have profound and mostly negative effects on plant growth and development. As a consequence, the phenology and distribution of crop and wild plants are already being affected by climate change (Willis et al., 2008). Plants have evolved various developmental responses to adapt to high temperature stresses One such response is acceleration of the vegetative to reproductive transition (flowering) (Balasubramanian et al., 2006), which increases species survival under high temperature stress, as dormant seeds have greater tolerance of stress than growing plants. Various morphological changes can occur under high temperature conditions: stem elongation, leaf hyponastic growth, and decrease in leaf thickness (Quint et al., 2016). These morphological changes are collectively described as thermomorphogenic responses, and are assumed to increase plant cooling capacity by enhancing leaf transpiration efficiency, thereby helping plants survive under high temperature stress (Crawford et al., 2012). Support for this belief has been obtained recently in a study that showed thermomorphogenic growth enhanced plant tolerance to heat stress (Zhu et al., 2016). Our understanding of thermomorphogenesis is still incomplete particularly with regard to the molecular mechanisms. Elucidation of the mechanisms of thermomorphogenesis will be of value for developing heat-tolerant crops, which may be critical in future for food security in a warming climate.

In *Arabidopsis*, the bHLH transcription factor PHYTOCHROME INTERACTING FACTOR4 (PIF4) is a key regulator of thermomorphogenesis (Koini et al., 2009; Choi and Oh, 2016; Quint et al., 2016). PIF4 was first identified as a phytochrome interacting factor that negatively regulates a light signaling pathway (Huq and Quail, 2002). The role of PIF4 in thermomorphogenesis was later indicated by the thermoinsensitive growth phenotypes of a *pif4* mutant (Koini et al., 2009). High temperatures reduce the activity of EARLY FLOWERING 3 (ELF3), a transcriptional repressor of *PIF4*; this reduction in ELF3 activity results in transcriptional activation of *PIF4* (Mizuno et al., 2014; Box et al., 2015). The increase in PIF4 at high temperature causes increased binding to the promoters of the auxin biosynthetic genes *YUC8* and *TAA1* and of the auxin responsive genes *IAA19* and *IAA29*, which directly activates their expression (Koini et al., 2009; Franklin et al., 2011; Oh et al., 2012; Sun et al., 2012). The resulting activated auxin biosynthesis/signaling pathway induces thermomorphogenic alterations including hypocotyl and petiole elongation (Franklin et al., 2011; Sun et al., 2012). In addition to auxin, plant hormones such as brassinosteroid and gibberellin have been reported to be required for thermomorphogenic growth (Gray et al., 1998; Stavang et al., 2009). It was recently shown that blue and UV-B light suppresses thermomorphogenesis by inhibiting PIF4 activity via multiple mechanisms (Ma et al., 2016; Pedmale et al., 2016; Hayes et al., 2017). The circadian clock has also been found to participate in thermomorphogenesis through the action of the evening-expressed circadian clock protein TOC1 (Zhu et al., 2016). TOC1 directly interacts with PIF4 and represses its activity, thereby suppressing thermomorphogenic growth during the evening (Zhu et al., 2016).

The homologous proteins LONGIFOLIA1 (LNG1) and LONGIFOLIA2 (LNG2) regulate leaf morphology in *Arabidopsis* (Lee et al., 2006). The LNG proteins positively promote longitudinal polar cell elongation; this role is exemplified by the *lng* gain-of-function mutant (*lng-D*), which displays extremely long leaf blades, elongated floral organs, and elongated siliques (Lee et al., 2006). In contrast, *lng1;lng2* double mutants have short petioles and leaf blades (Lee et al., 2006). Consistent with their physiological functions, *LNG1* and *LNG2* genes are expressed in various organs including the petioles, leaf blades, flowers, and roots (Lee et al., 2006). The *LNGs* encode plant specific proteins without any known functional domains. The LNG proteins were detected in both the cytosol and nucleus (Lee et al., 2006), although the molecular mechanisms of LNG-mediated longitudinal polar cell elongation are still unknown. LNG gene family was identified in the plant species including in moss, *Physcomitrella patens*, from the database^1^ (Tello-Ruiz et al., 2016). The *Arabidopsis* genome contains two other LNG homologous proteins, LNG3 (AGI:At1g74160) and LNG4 (AGI:At1g18620). In addition to the regulation of longitudinal polar cell elongation, LNG proteins have been shown to be involved in microtubule organization through the recruitment of TONNEAU1 (TON1) to the cytoskeleton (Drevensek et al., 2012).

Here, we show that PIF4 directly binds to the promoters of *LNG1* and *LNG2*. qRT-PCR analyses revealed that expression of *LNG1* and *LNG2* was temperature-regulated by PIF4 dependent manner. We further show that LNG proteins are required for the high temperature activation of the auxin biosynthetic gene *YUC8* and auxin responsive gene *IAA29*. Thermomorphogenic growth is compromised in the *lng* quadruple (*lngq*) mutant that carries mutations in all four *LNG* genes. Together, our results demonstrate that PIF4 transcriptionally activates *LNG1* and *LNG2* in response to high temperature, and thereby induces thermomorphogenic growth.

## Materials and Methods

### Plant Materials and Growth Conditions

*Arabidopsis thaliana* plants were grown in a greenhouse with 16 h light/8 h dark cycles at 22–24°C for general growth and seed harvesting. All *A. thaliana* plants used in this study belonged to the Col-0 ecotype background. The *PIF4p::PIF4-MYC* transgenic plants used in the ChIP assays were described previously (Oh et al., 2012). The *lngq* plants were generated by crossing the single *lng* mutants *lng1-3* (Salk_135586), *lng2-1* (Salk_067658), *lng3-2* (Salk_068678), and *lng4-1* (Salk_144569).

### Hypocotyl Length Measurement

Seeds were sterilized in 70% (v/v) ethanol and 0.01% (v/v) Triton X-100 and then plated on MS medium (Duchefa) supplemented with 0.75% phyto agar (Duchefa). After 3 days of stratification at 4°C, the plates were placed under white light for 6 h to promote seed germination and incubated at 20°C under 24 h light conditions (light intensity: 30 μmol m^-2^ s^-1^) for 7 days or incubated at 20°C for 4 days followed by incubation at 28°C for 3 days. Seven-day-old seedlings were photocopied and hypocotyl lengths were measured using ImageJ software^2^.

### Petiole Length Measurement

WT and *lngq* seedlings were grown under white light at 20°C for 2 weeks or at 20°C for 1 week followed by incubation at 28°C for 1 week. Two-week-old plants were photocopied and petiole lengths (from the base of leaf blade to the point of attachment to hypocotyl/shoot apical meristem) were measured using ImageJ software^3^.

### qRT-PCR Gene Expression Analysis

Seedlings were grown at 20°C in 12 h light/12 h dark conditions for 4 days and then transferred to 24 h light conditions on the 5th day. The seedlings were then incubated at 20°C for 24 h or subjected to 28°C during ZT20 to ZT24, before harvesting for total RNA extraction. Total RNA was extracted from the seedlings using the MiniBEST Plant RNA extraction kit (TaKaRa). M-MLV reverse transcriptase (Fermentas) was used to synthesize cDNA from the RNA. Quantitative real-time PCR (qRT-PCR) was performed using the CFX96 Real-Time PCR detection system (Bio-Rad) and the EvaGreen master mix (Solgent). Gene expression levels were normalized to that of the SERINE/THREONINE PROTEIN PHOSPHATASE 2A (*PP2A*) gene and are shown relative to the expression levels in wild type. Gene specific primers are listed in Supplementary Table S1.

### Chromatin Immunoprecipitation (ChIP) Assays

Chromatin Immunoprecipitation assays were performed as previously described (Oh et al., 2014). Seven-day-old *PIF4p::PIF4-MYC* seedlings were placed in 1% formaldehyde under vacuum for 20 min to produce protein-DNA cross-links. Chromatin was recovered from the plant cells and immunoprecipitated as described [21]; the chromatin was resuspended in lysis buffer (50 mM Tris–HCl pH 8.0, 10 mM EDTA, 200 mM NaCl, 0.5% Triton X-100, 1 mM PMSF) and sheared by sonication to reduce the average DNA fragment size to around 300 to 500 bps. The sonicated chromatin complex was then immunoprecipitated by an anti-MYC antibody (Cell Signaling Technology) bound to protein A agarose beads (Millipore). The beads were washed with low-salt buffer (50 mM Tris–HCl at pH 8.0, 2 mM EDTA, 150 mM NaCl, 0.5% Triton X-100), high-salt buffer (50 mM Tris–HCl at pH 8.0, 2 mM EDTA, 500 mM NaCl, 0.5% Triton X-100), LiCl buffer (10 mM Tris–HCl at pH 8.0, 1 mM EDTA, 0.25 M LiCl, 0.5% NP-40, 0.5% deoxycholate), and TE buffer (10 mM Tris–HCl at pH 8.0, 1 mM EDTA) and eluted with elution buffer (1% SDS, 0.1 M NaHCO_3_). The PIF4-MYC-bound DNA fragments were purified using a PCR purification kit (Thermo Scientific) and analyzed by ChIP-qPCR. The enrichment of DNA was normalized to that of *PP2A.* Primers for ChIP-qPCR are listed in Supplementary Table S1.

### Protein Extraction and Western Blot Analysis

Wild type and *lngq* mutant seedlings were grown in the same conditions as for the qRT-PCR tests. Total proteins were extracted with 2x protein extraction buffer (100 mM Tris–HCl, pH 6.8, 25% glycerol, 2% SDS, 0.01% bromophenol blue, with β-mercaptoethanol added to 10% before use). Western blot analysis was performed to determine endogenous PIF4 levels using anti-PIF4 antibody (Agrisera, AS16 3157).

## Results and Discussion

### PIF4 Directly Binds to the Promoters of *LNG1* and *LNG2*

**FIGURE 1:**
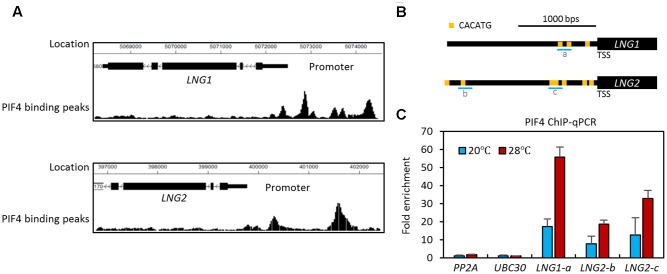
PHYTOCHROME INTERACTING FACTOR4 directly binds to the promoters of *LNG1* and *LNG2.* **(A)** Previous ChIP-Seq analyses (Oh et al., 2012) showing the PIF4 binding peaks around the promoters of *LNG1* and *LNG2*. **(B)** A diagram of the *LNG1* and *LNG2* promoters showing the positions of PBE (PIF4 Binding E-box: CATGTG) motifs. a, b, and, c indicate the ChIP amplicons in the ChIP assays in **(C)**. **(C)** ChIP-quantitative PCR assays of PIF4 binding to *LNG1* and *LNG2* promoters. Five-day-old WT and *PIF4p::PIF4-MYC* transgenic seedlings were grown at 20°C, then switched to 28°C for 4 h or kept at 20°C; the seedlings were then harvested for ChIP assays using an anti-MYC antibody. Enrichment of DNA was calculated as the ratio between *PIF4p::PIF4-MYC* and WT control, normalized to that of the *PP2A* coding region as an internal reference. Error bars indicate standard deviation (SD, *n* = 3).

Our previous ChIP-Seq analyses identified *LNG1* and *LNG2* as PIF4 target genes (Oh et al., 2012). As shown in **Figure 1A**, there are several PIF4 binding peaks in the promoters of both *LNG1* and *LNG2*, indicating that PIF4 directly binds to the promoter of these genes. In addition, *LNG1* was identified as a PIF3 target gene and both *LNG1* and *LNG2* were identified as PIF5 target genes by PIF3 and PIF5 ChIP-Seq analyses, respectively (Hornitschek et al., 2012; Zhang et al., 2013). *LNG3* and *LNG4* have also been identified as PIF1 and PIF4 target genes (Supplementary Figure S1A; Oh et al., 2014; Pfeiffer et al., 2014). To confirm the results of the previous large scale experiments, we performed ChIP assays with transgenic plants expressing a PIF4-Myc fusion protein under a *PIF4* promoter (*PIF4p::PIF4-MYC*). To examine the effect of high temperature, we included seedlings exposed for 4 h to high temperature. The ChIP assays showed that PIF4 directly binds to the promoter of *LNG1* (about -400 bps from the transcription start sequence, TSS) and the promoter of *LNG2* (about -600 bps and -1700 bps from the TSS) (**Figures 1B,C**). These results are consistent with those of the previous PIF4 ChIP-Seq analyses. PIF4 appears to bind to these promoters through the E-box motif variant (CACATG), which has previously been shown to be a PIF4 binding motif (Oh et al., 2012; Zhang et al., 2013). Interestingly, PIF4 binding to these promoters was increased by the high temperature treatment (**Figure 1C**); this is consistent with previous studies showing that PIF4 protein activity is increased at elevated temperatures (Mizuno et al., 2014; Zhu et al., 2016). Our results indicate that PIF4 directly binds to the promoters of both *LNG1* and *LNG2*, and presumably regulates their expression in a temperature dependent manner.

### The Expression of *LNG1* and *LNG2* Is Increased by High Temperature

**FIGURE 2:**
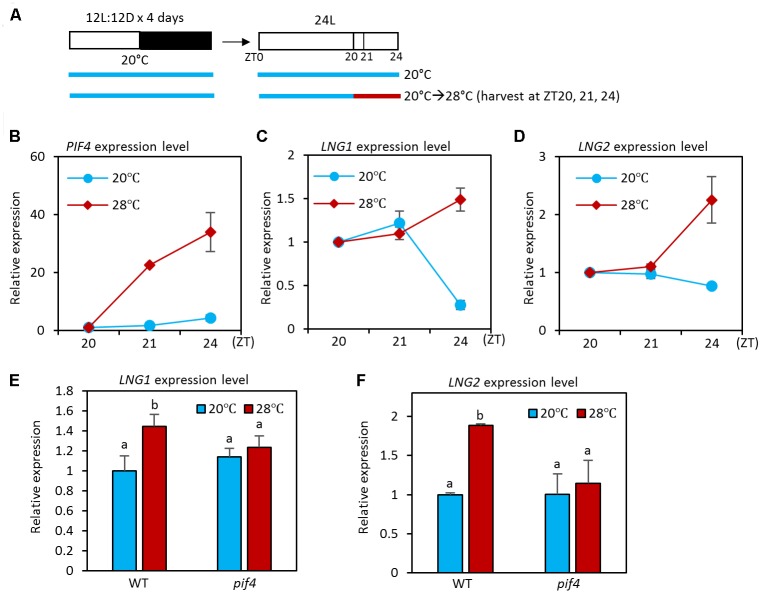
High temperature activates the expression of *LNG1* and *LNG2* in a PIF4 dependent manner. **(A)** Diagram showing plant growth conditions used for the qRT-PCR analyses in **(B)** and **(F)**. Seedlings were maintained under 12 h light/12 h dark conditions at 20°C for 4 days, and then transferred to 24 h light conditions. The seedlings were then exposed for 1 or 4 h to high temperature (28°C) from ZT20 and harvested for RNA extraction. **(B–D)** The qRT-PCR analysis of the expression levels of *PIF4*, *LNG1*, and *LNG2* in WT seedlings under the indicated growth conditions. Gene expression levels were normalized to *PP2A* and presented as values relative to those of the WT seedlings at 20°C at ZT20. Error bars indicate standard deviation (SD, *n* = 3). **(E,F)** The qRT-PCR analysis of the expression levels of *LNG1* and *LNG2*. WT and *pif4* mutant seedlings were exposed for 4 h to high temperature (28°C) from ZT20 or kept at 20°C. Error bars indicate SD (*n* = 3). Different letters above each bar indicate statistically significant differences (ANOVA and Tukey’s HSD; *P* < 0.05).

PHYTOCHROME INTERACTING FACTOR4 is a key regulator of thermoresponsive growth and thermoregulation of expression of various genes (Choi and Oh, 2016; Quint et al., 2016). In addition, PIF4 activity increases as ambient temperature is elevated (Koini et al., 2009). Since the PIF4 transcription factor activates transcription and binds directly to *LNG1* and *LNG2* promoters (Zhu et al., 2016) and the binding is enhanced at high temperatures (**Figure 1**), it is highly likely that the expression of *LNG1* and *LNG2* is also temperature-regulated. To test whether this is the case, we used qRT-PCR to determine the transcriptional responses of *LNG1* and *LNG2* to an increase in ambient temperature. For the qRT-PCR analyses, seedlings were grown under 12 h light/12 h dark conditions at 20°C for 4 days, and then transferred to 24 h light conditions (**Figure 2A**). The seedlings were exposed for 1 or 4 h to a temperature of 28°C at ZT20 at which time the plants are highly sensitive to high temperature due to the elevated PIF4 activity (Mizuno et al., 2014). The qRT-PCR analyses showed that expression of both *LNG1* and *LNG2* was not significantly affected by 1 h of high temperature treatment (**Figures 2C,D**). However, while *LNG1* expression was decreased at ZT24 in the seedlings at 20°C, it was slightly increased in the seedlings at 28°C (**Figures 2C,D**). *LNG2* expression was also induced by 4 h of high temperature treatment. In contrast, *PIF4* expression was rapidly induced in response to high temperature within 1 h (**Figure 2B**). The rapid activation of *PIF4* before *LNGs* induction suggests that PIF4 mediates the high temperature-induced *LNG1* and *LNG2* expression.

### PIF4 Mediates the High Temperature Activation of *LNG1* and *LNG2* Expression

Next, we examined whether high temperature activation of *LNG1* and *LNG2* requires PIF4 activity. To perform this analysis, we measured the expression of *LNG1* and *LNG2* in wild type and *pif4* mutant grown under normal conditions or at 28°C for 4 h (**Figure 2A**). Compared to wild type plants, expression of *LNG1* and *LNG2* was not significantly altered by high temperature in the *pif4* mutant (**Figures 2E,F**), suggesting that PIF4 mediates the high temperature activation of *LNG1* and *LNG2* expression. Since the PIF4 binding to the *LNG1* and *LNG2* promoters is increased at high temperatures (**Figure 1C**), these results support our hypothesis that the elevation in the ambient temperature increases PIF4 activity and binding to the promoters of *LNG1* and *LNG2*, which leads to the transcriptional activation of *LNG1* and *LNG2*.

However, *LNG1* and *LNG2* expression was not significantly altered in the *pif4* mutant at 20°C. Since other PIFs (including PIF1, PIF3, and PIF5) have also been shown to directly bind to *LNG1* and *LNG2* promoters, it is possible that the remaining PIFs redundantly regulate *LNG1* and *LNG2* in the *pif4* mutant at 20°C. In support of this possibility, previous microarray analyses with a *pif4;pif5* double mutant showed that both *LNG1* and *LNG2* expression is lower in the *pif4;pif5* double mutant than in the wild type at normal temperatures (Supplementary Figure S2) (Hornitschek et al., 2012).

### LNGs Are Required for the Thermoresponsive Growth

**FIGURE 3:**
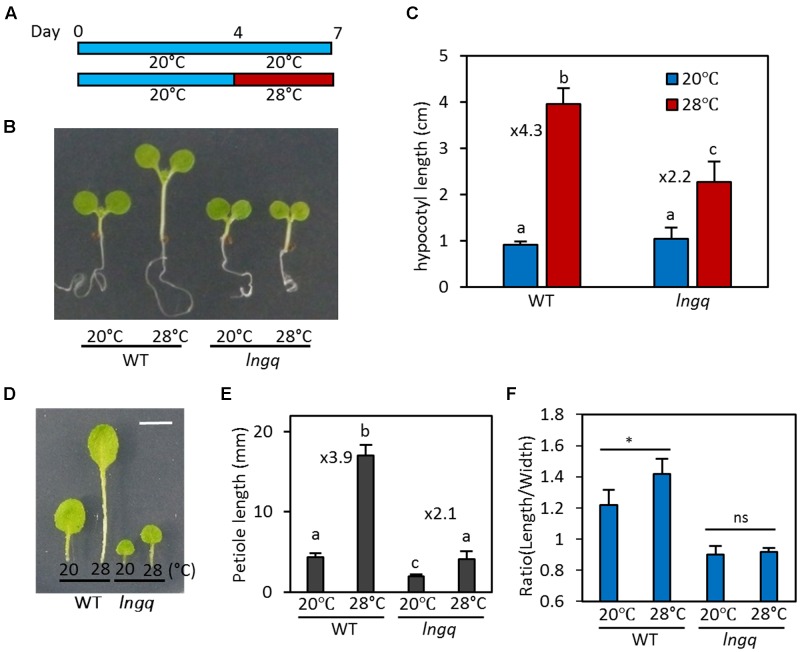
LONGIFOLIA activities are required for thermomorphogenic growth. **(A)** Diagram showing plant growth conditions for hypocotyl length measurements. WT and *lngq* seedlings were grown at 20°C for 7 days or grown at 20°C for 4 days followed by incubation at 28°C for 3 days before harvesting for hypocotyl length measurements. **(B)** Representative seedlings grown under the conditions described in **(A)**. **(C)** Average hypocotyl lengths of WT and *lngq* seedlings grown under the conditions described in **(A)**. Error bars indicate standard deviation (SD, *n* = 10 plants). Numbers indicate ratios of hypocotyl lengths (28°C/20°C). Different letters above each bar indicate statistically significant differences (ANOVA and Tukey’s HSD; *P* < 0.05). **(D)** Representative leaf blades and petioles of wild type and *lngq* plants grown at 20°C or 28°C for 2 weeks. Bar indicates 5 mm. **(E)** Average petiole lengths of WT and *lngq* plants grown at two different temperatures. Error bars indicate SD (*n* = 20 petioles). Numbers indicate ratios of petiole lengths (28°C/20°C). Different letters above each bar indicate statistically significant differences (ANOVA and Tukey’s HSD; *P* < 0.05). **(F)** Average ratio of the leaf length to width of plants grown at 20°C or 28°C. The third and fourth leaves were analyzed. Error bars indicate SD (*n* = 10 leaves). ^∗^*P* < 0.05 (Student’s *t*-test) and ns indicates not significant.

Since both *LNG1* and *LNG2* are directly regulated by PIF4 and high temperature, it is likely that these LNG proteins are involved in PIF4-mediated developmental responses including thermomorphogenesis. Therefore, we examined whether the LNG proteins play a role in thermomorphogenesis. To perform this analysis, wild type and *lng* quadruple mutant (*lng1;lng2;lng3;lng4*, *lngq*) seedlings were grown at 20°C for 7 days or at 20°C for 4 days followed by 28°C incubation for 3 days before harvesting for hypocotyl measurement (**Figure 3A**). We analyzed the *lngq* mutant in order to completely remove any residual activities of other LNG proteins because *LNG3* and *LNG4* are also direct target genes of PIF4 (Supplementary Figures S1A,B), and *LNG4* expression is increased at high temperatures (Supplementary Figure S1C). Under these growth conditions, hypocotyl elongation in wild type seedlings was strongly promoted by the high temperature (28°C). The hypocotyls of wild type at 28°C were four times longer than those of wild type seedlings grown at 20°C (**Figures 3B,C**). However, the hypocotyl length of *lngq* seedlings at 28°C was only about twofold longer than that of those grown at 20°C (**Figures 3B,C**), indicating that hypocotyl growth in *lngq* seedlings is less sensitive to high temperature than in wild type seedlings. In addition to the hypocotyls, leaf petiole elongation is also promoted by high temperature, which is mediated by PIF4 (Koini et al., 2009). The petiole elongation response to high temperature was also attenuated in the *lngq* plants, similar to the hypocotyl elongation (**Figures 3D,E**). These results suggest that PIF4 induces thermoresponsive growth by activating *LNGs* as well as auxin genes.

Previous studies showed that LNG1 and LNG2 promote longitudinal polar cell elongation, thereby determining leaf morphology (Lee et al., 2006). Since these *LNGs* are transcriptionally activated at high temperatures, it is likely that leaf morphology would be changed in response to an increase in ambient temperature. Indeed, wild type plants grown at 28°C have more elongated leaf blade (high ratio of leaf length to leaf width) than the same plants grown at 20°C (**Figures 3D,F**). In contrast to wild type, leaf shapes of *lngq* mutant plants were not significantly altered by high temperature (**Figure 3F**), indicating that high temperatures promote the elongation of leaf blades through the transcriptional activation of *LNGs*.

### LNG Proteins Support Thermoregulation of Auxin Biosynthetic and Signaling Genes

**FIGURE 4:**
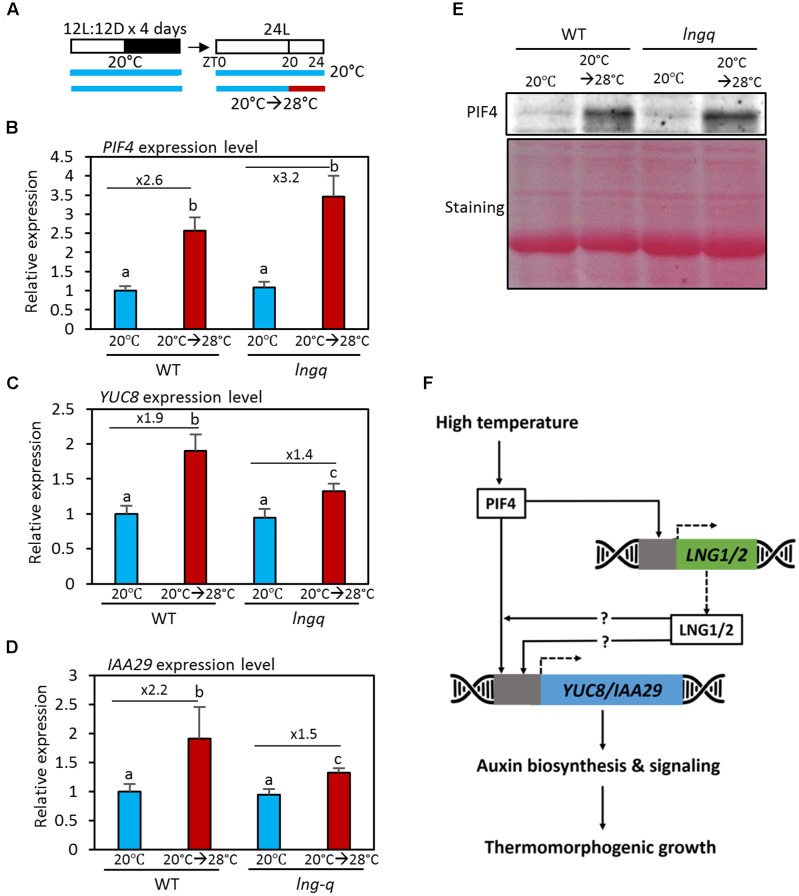
The expression of *YUC8* and *IAA29* is hyposensitive to high temperature in *lngq* seedlings. **(A)** Diagram showing plant growth conditions used for the qRT-PCR analyses in **(B–D)**. Seedlings were maintained under 12 h light/12 h dark at 20°C for 4 days, and then transferred to 24 h light conditions. The seedlings were then exposed for 4 h to high temperature (28°C) at ZT20 and harvested for RNA extraction. **(B–D)** The qRT-PCR analysis of the expression level of *YUC8* and *IAA29*. Gene expression levels were normalized to *PP2A* and presented as values relative to those of the WT seedlings at 20°C. Error bars indicate standard deviation (SD, *n* = 3). Numbers indicate ratios of the expression levels (28°C/20°C). Different letters above each bar indicate statistically significant differences (ANOVA and Tukey’s HSD; *P* < 0.05). **(E)** Western blotting with anti-PIF4 antibody showed that PIF4 protein levels were increased by high temperatures in both wild type and *lngq* seedlings. Total protein was extracted from the seedlings grown in the conditions described in **(A)**. Equal loading of samples is shown by Ponceau S staining. **(F)** A hypothetical model depicting PIF4-LNGs-mediated thermomorphogenic growth. At elevated temperatures, PIF4 transcription factor binds to the promoters of *LNG1* and *LNG2* as well as auxin-related genes (*YUC8* and *IAA29*), and activate their expression. The increased LNG proteins further activate the expression of *YUC8* and *IAA29* through unknown mechanisms. The resulting increased auxin biosynthesis and signaling induce thermomorphogenic growth.

At high temperatures, PIF4 increases endogenous auxin levels by directly activating the auxin biosynthetic genes *YUC8*, *TAA1*, and *CYP79B*. PIF4 also directly activates the expression of the auxin responsive genes *IAA19* and *IAA29* (Koini et al., 2009; Franklin et al., 2011; Oh et al., 2012; Sun et al., 2012). To examine the molecular mechanisms in which LNG proteins participate in the high temperature responses, we determined the expression levels of *PIF4* and its target genes *YUC8* and *IAA29* in wild type and *lngq* seedlings (**Figure 4A**). Previous studies showed that *PIF4* is transcriptionally activated in response to high temperature (Koini et al., 2009; Yamashino et al., 2013). Consistent with this finding, the expression of *PIF4* was found here to increase after a high temperature treatment in wild type seedlings (**Figure 4B**). *PIF4* expression was also significantly upregulated by high temperature in *lngq* seedlings (**Figure 4B**), showing that the reduced thermomorphogenic growth of these seedlings is not caused by a reduced *PIF4* level. Expression of the PIF4 target genes *YUC8* and *IAA29* expression was significantly elevated after high temperature in wild type seedlings (**Figures 4C,D**). However, the upregulation of *YUC8* and *IAA29* expression was not as great in *lngq* seedlings (**Figures 4C,D**). These results indicate that LNG proteins mediate in part the high temperature activation of auxin biosynthetic (*YUC8*) and responsive genes (*IAA29*). The expression patterns of *YUC8* and *IAA29* are consistent with the lack of hypocotyl elongation under high temperature conditions in *lngq* seedlings (**Figure 3**), suggesting that the reduced auxin biosynthesis contributes to the hypocotyl elongation defect of *lngq* seedlings grown at high temperatures.

## Conclusion

Here, we demonstrated that the homologous proteins LNG1 and LNG2 regulate thermomorphogenic growth. At an elevated temperature, PIF4 transcription factor directly binds to the promoters of both *LNG1* and *LNG2* and activates their expression. The increased level of LNG proteins then induces thermomorphogenic growth including hypocotyl, petiole, and leaf blade elongation at least partly through up-regulation of auxin biosynthetic and responsive genes (*YUC8* and *IAA29*). Since *YUC8* and *IAA29* are known to be directly regulated by PIF4, LNGs are likely to increase the PIF4 level to induce *YUC8* and *IAA29* expression. However, *PIF4* mRNA expression and PIF4 protein levels were not significantly affected in *lngq* seedlings (**Figures 4B,E**), suggesting that LNGs activate PIF4 post-translationally either by promoting PIF4 binding to target DNAs or enhancing PIF4 transcription activity. In addition, PIF4 and LNGs appear to constitute a feed-forward loop because the PIF4-activated LNGs potentiate PIF4 activation of auxin pathway genes (*YUC8* and *IAA29*). Such a feed-forward loop may enable plants to rapidly respond to an ambient temperature increase (**Figure 4F** and Supplementary Figure S3).

It has previously been shown that the LNG proteins regulate microtubule organization by recruiting a centrosomal protein (Drevensek et al., 2012); however, the mechanism through which LNG proteins control gene expression remains to be elucidated. A previous study suggested that the elongated petioles and leaf elevation may potentially enhance leaf transpiration rates by increasing the diffusion of water vapor from stomata (Crawford et al., 2012). It would be also of interest to examine whether LNG-induced leaf morphological changes also contribute to enhanced leaf transpiration rates at high temperatures.

## Author Contributions

GH, J-YZ, YL, and EO designed research, analyzed data, wrote manuscript, assembled and edited figures. SK designed experiments, conducted research, analyzed data. TN conducted experiments. JK conducted research and analyzed data.

## Conflict of Interest Statement

The authors declare that the research was conducted in the absence of any commercial or financial relationships that could be construed as a potential conflict of interest.

## Footnotes

1 http://www.gramene.org/

2 http://rsb.info.nih.gov/ij

3 http://rsb.info.nih.gov/ij
