# Supplementary figures and images for: PIF4 Promotes Expression of LNG1 and LNG2 to Induce Thermomorphogenic Growth in Arabidopsis
Source: Front Plant Sci. 2017 Jul 25;8:1320. doi: 10.3389/fpls.2017.01320 (PMC5524824; doi:10.3389/fpls.2017.01320)

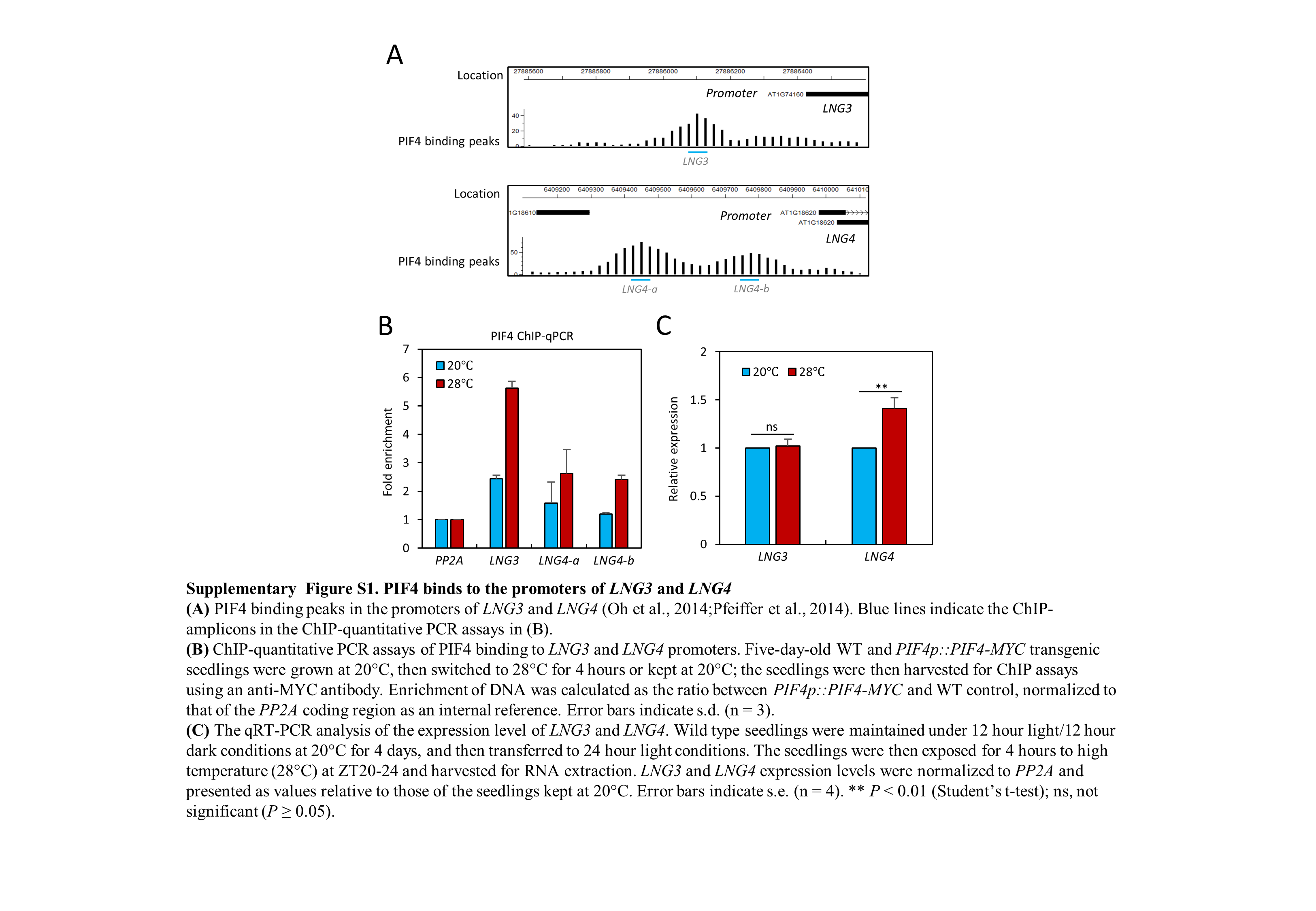

Supplement: Supplementary file 2 [file Image_1.TIF]

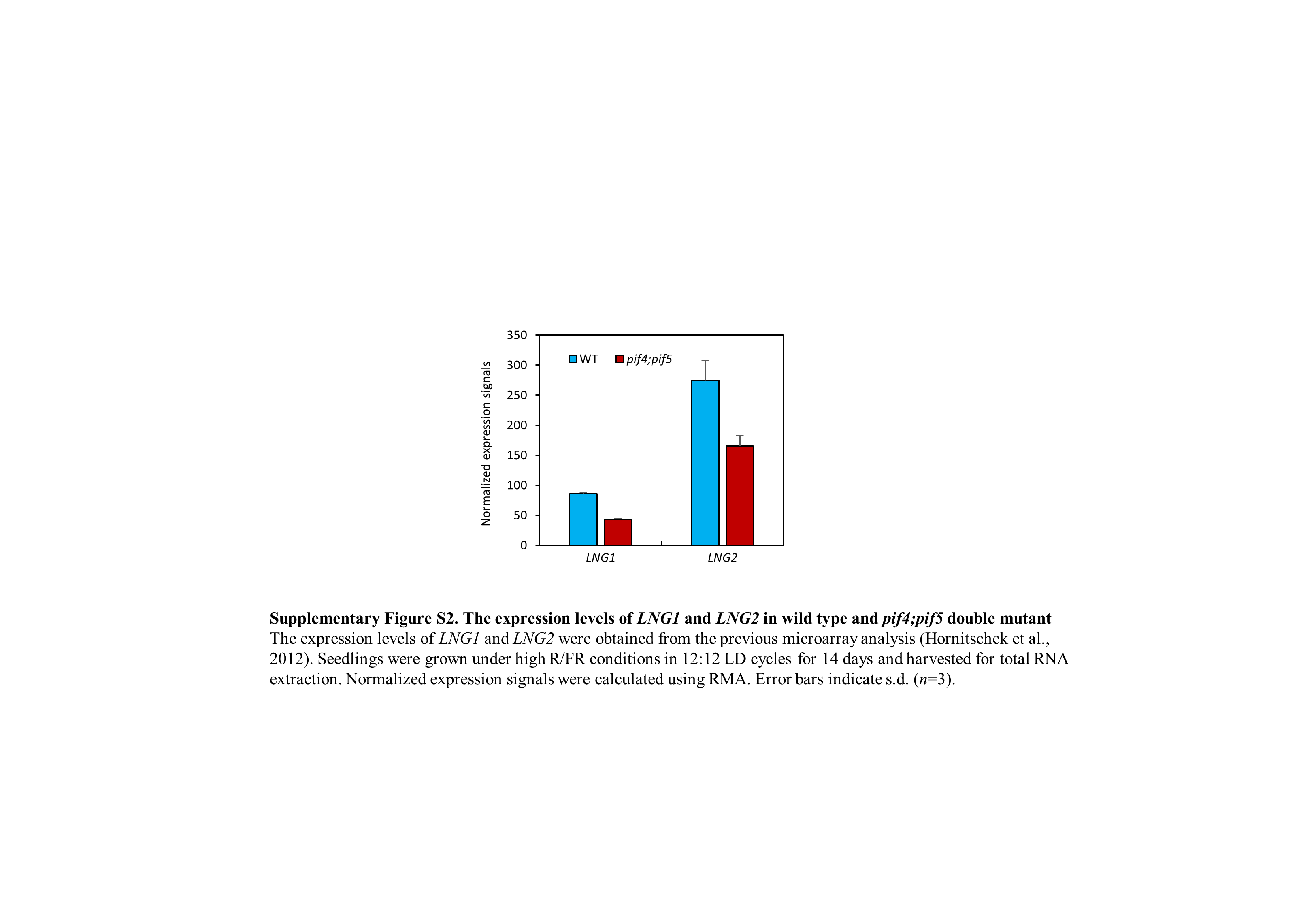

Supplement: Supplementary file 3 [file Image_2.TIF]

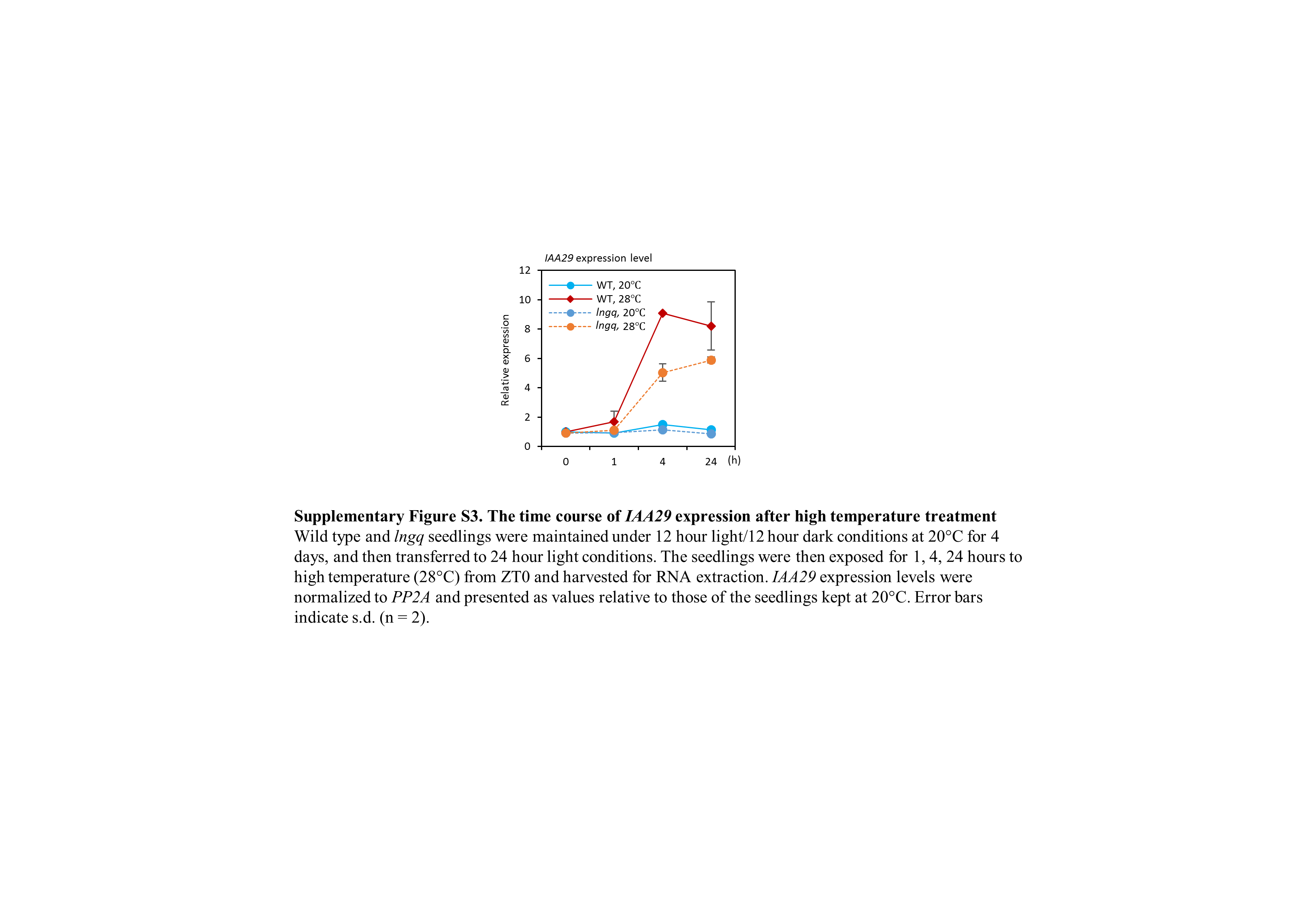

Supplement: Supplementary file 4 [file Image_3.TIF]
